# Anatomic Shoulder Arthroplasty: Technical Considerations

**DOI:** 10.2174/1874325001711011115

**Published:** 2017-09-30

**Authors:** Bogdan A. Matache, P. Lapner

**Affiliations:** 1 Orthopaedic Surgery Resident, Division of Orthopaedic Surgery, University of Ottawa, Ottawa, Ontario, Canada; 2 Associate Professor of Surgery, Division of Orthopaedic Surgery, University of Ottawa, Ottawa Hospital Research Institute, Ottawa, Ontario, Canada

**Keywords:** Glenohumeral arthritis, Glenoid bone loss, Lesser tuberosity osteotomy, Midsubstance tenotomy, Reverse total shoulder arthroplasty, Subscapularis peel, Subscapularis-preserving, Total shoulder arthroplasty, Type B2 glenoid

## Abstract

Osteoarthritis of the shoulder is a common condition in the aging population, and it can have profound effects on patients’ quality of life. The anatomic total shoulder arthroplasty is a well-described treatment modality resulting generally excellent outcomes. The objective of this review is to discuss the technical aspects of primary anatomic total shoulder arthroplasty, and to provide a framework to follow to achieve a successful surgical result. The topics covered include preoperative planning, surgical considerations, and approaches, humeral preparation, glenoid bone loss and the emerging concept of using the reverse total shoulder arthroplasty for the type B2 glenoid.

## 1 INTRODUCTION

Glenohumeral arthritis is a common condition in the aging population, and it can have profound effects on patients’ quality of life. Total shoulder arthroplasty (TSA), hemiarthroplasty, and reverse total shoulder arthroplasty (RTSA) have all been well-described in the context of shoulder osteoarthritis, and the number of these procedures has been steadily rising over the past decade. This can be attributed to an increasingly elderly population, improved surgical implant technology, and increased surgeon familiarity with the procedure resulting in overall excellent outcomes (>90%) [1, 2]. For example, between 2000 and 2008, the annual number of shoulder arthroplasties increased 2.5-fold, with an 11% rise in the elderly population (over the age of 65), and an increase in the number of implant manufacturers to eight [3]. The primary goals of shoulder arthroplasty are to provide pain relief, stability, and restore motion [4]. These may be achieved through a thorough understanding of the indications for surgery, indications for each prosthetic type, and proper technique. The purpose of this article is focus on the technical aspects of primary anatomic TSA, and aim to provide a framework to follow to achieve a successful surgical result.

## 2 PREOPERATIVE PLANNING

**Fig. (1):**
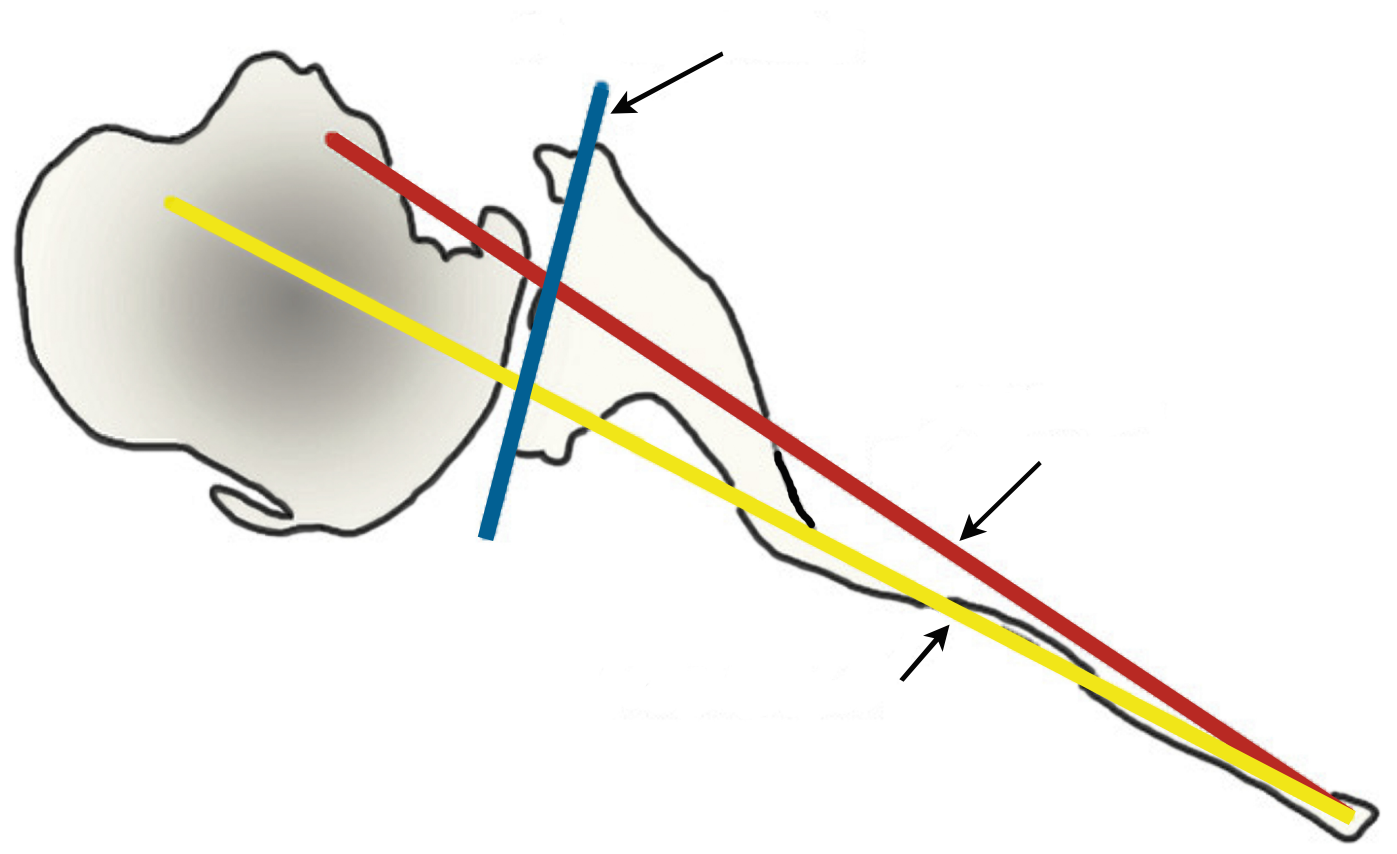
Graphic depicting the Friedman (red), scapula body (yellow) and intermediate (blue) line methods of determining scapular version. (Adapted from Rouleau DM, Kidder JF, Pons-Villanueva J, Dynamidis S, Defranco M, Walch G: Glenoid version: How to measure it? Validity of different methods in two-dimensional computed tomography scans. J Shoulder Elbow Surg 2010;19 [8]:1230-1237.).

**Fig. (2):**
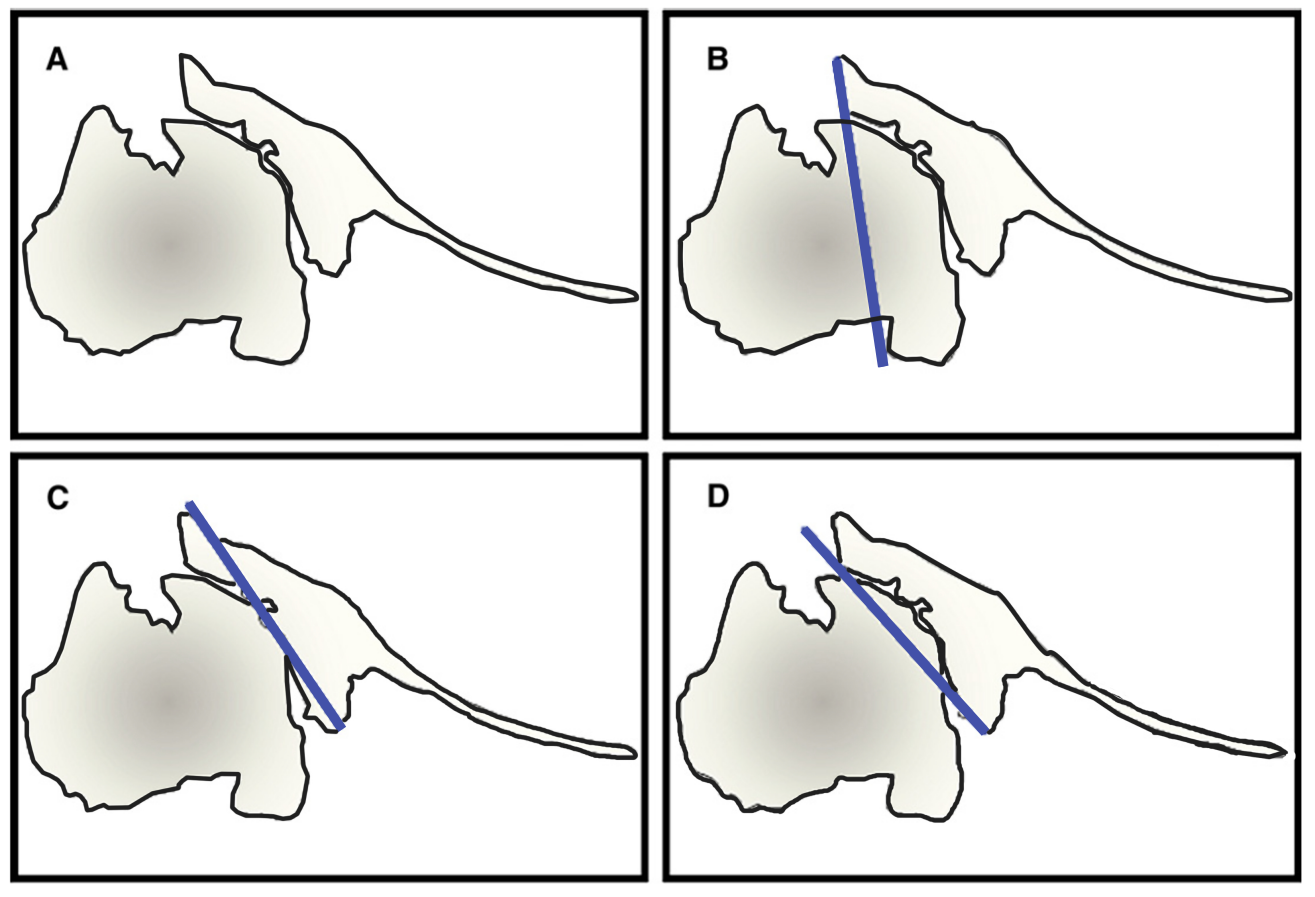
Image depicting a type B2 glenoid **(A)** With three reference lines described by Walch: **(B)** The paleoglenoid, which represents the native glenoid version, **(C)** Intermediate glenoid, and **(D)** The neoglenoid, representing the new version of the glenoid. (Adapted from Rouleau DM, Kidder JF, Pons-Villanueva J, Dynamidis S, Defranco M, Walch G: Glenoid version: How to measure it? Validity of different methods in two-dimensional computed tomography scans. J Shoulder Elbow Surg 2010;19 [8]:1230-1237.).

Unconstrained TSA is indicated in the treatment of degenerative or inflammatory conditions affecting the shoulder joint, including primary osteoarthritis (OA), avascular necrosis (AVN), rheumatoid arthritis (RA), and post-traumatic arthritis (PTA) when the rotator cuff is intact [5]. Glenoid component loosening is the most common complication following TSA, and is often due to technical errors leading to component malposition, which increases stress forces across the glenoid component [2, 6-8]. A careful pre-operative evaluation is necessary in all cases, including standard radiographs, including true AP in neutral, internal, and external rotation, and axillary views of the shoulder. The true AP views are useful to assess bone quality, presence and location of osteophytes, humeral canal diameter for templating, as well as neck/shaft angle, and humeral head diameter [6, 9]. The axillary view is used to recognize posterior glenoid wear and retroversion, although it may overestimate these in most cases [10]. Two- or three-dimensional CT scan may be used to better characterize glenoid version by using Friedman’s line, drawn from the medial tip of the scapula body to the midpoint of the glenoid fossa (Fig. **1**) [11, 12]. A line perpendicular to this drawn from the anterior edge of the glenoid fossa defines neutral version; if the posterior edge of the glenoid fossa lies medial to this line, it is retroverted. Rouleau **et al*.* [11] more recently described the paleoglenoid (line along the high, anterior glenoid), intermediate glenoid (connecting the anterior and posterior glenoid edge), and neoglenoid (along the worn, posterior glenoid) reference lines, and found that the intermediate glenoid line is more reliable at predicting version in type B2 glenoids, discussed below (Fig. **2**). Other techniques, such as the scapula body line described elsewhere, have also been used to predict glenoid version [12]. MRI may be indicated when a rotator cuff tear is suspected, as well as in patients with rheumatoid arthritis of the shoulder, who are known to have a high incidence of atraumatic cuff tears [12, 13].

Humeral head subluxation (HHS) is another factor that should be recognized preoperatively, as higher degrees of preoperative subluxation correlate to elevated rates of failure [14, 15]. Subluxation may recur postoperatively and can contribute to early glenoid component loosening [16, 17]. The Papilion and Shall method is the most accepted measure of HHS, and it involves comparing the center of the humeral head with the midpoint of a line connecting the anterior and posterior edges of the glenoid to determine if the head is subluxated anteriorly or posteriorly [18]. This technique was described using fluoroscopy, and was later adapted by Walch **et al*.* [19] to account for glenoid types with significant posterior wear. They determined a more accurate measure of HHS on two-dimensional (2D) CT scan by referencing the humeral head diameter to Friedman’s line. In this method, the axial cut demonstrating the widest anterior-to-posterior humeral head diameter is selected, and the percentage of humeral head posterior to Friedman’s line is measured. Posterior HHS greater than 55% is defined as subluxated. Furthermore, it has been suggested that HHS is multi- rather than unidirectional. Given than 2D CT only allows for assessment of subluxation in one plane, three-dimensional (3D) CT may in fact be more accurate at detecting out-of-plane HHS [20, 21]. 

## 3 SURGICAL CONSIDERATIONS

### 3.1 Approaches

#### 3.1.1 Deltopectoral Approach

The standard approach for surgical exposure during shoulder arthroplasty is the deltopectoral approach [22, 23]. The patient is positioned in a beach-chair position with the back at 30° to 45°, with all pressure points well-padded. The patient is positioned such that the shoulder hangs free over the edge of the operating table, allowing for unobstructed range of motion of the shoulder. The arm is then free-draped, and may be secured to a mechanical arm-holding device [9].

Once the interval is identified, the cephalic vein, may be taken either medially or laterally, depending on surgeon preference. Some surgeons advocate lateral retraction of the vein to preserve the larger tributaries from the deltoid muscle [9, 24]. Care should be taken to avoid injuring the coracoacromial ligament at the most proximal aspect of the exposure since its disruption can lead to anterosuperior subluxation of the humerus. The deltoid is retracted with a blunt Holman directly on bone so as to avoid injury to the axillary nerve. Over-retraction of the conjoint tendon is avoided to prevent injury to the musculocutaneous nerve which occurs as proximal as 3.8 cm from the tip of the coracoid process. The anterior circumflex vessels that cross the surgical field along the lower aspect of the subscapularis tendon are ligated or coagulated [25].

The long head of biceps (LHB) can be a significant source of anterior shoulder pain following shoulder arthroplasty [26, 27]. In a retrospective analysis from France, of 688 shoulder arthroplasty cases with a primary diagnosis of primary OA, 121 underwent concomitant biceps tenodesis at arthroplasty for an indication of abnormal appearing biceps tendon [28]. The tenodesis technique was standardized between surgeons, and consisted of resection of the proximal 5 cm of LHB, and suturing of the remaining stump to the humeral insertion of pectoralis major. Patients who underwent biceps tenodesis at the time of arthroplasty demonstrated significantly higher postoperative activity scores, shoulder mobility, and Constant scores at greater than 3 year follow-up without evidence of increased radiographic loosening or proximal humeral head migration.

A prospective cohort analysis of 140 consecutive cases treated with total shoulder arthroplasty for primary OA, post-traumatic OA, and rheumatoid arthritis [29]. LHB tenodesis was performed in approximately 40% of cases, either due to routine practice or abnormal appearance of the tendon during surgery. Among treatment successes, defined by a Constant score greater than 80/100, 54% received biceps tenodesis compared to 33% among the treatment failures. The odds ratio of a concomitant LHB tenodesis with treatment success was 2.38, which was statistically significant. A more recent prospective randomized trial followed 45 patients with four-part proximal humerus fractures, fracture dislocations, or head-splitting fractures treated with hemiarthroplasty, with and without LHB tenodesis [30]. At approximately two year follow-up there was a statistically significant difference in the modified Constant score, and in the occurrence of shoulder pain between the two groups favoring biceps tenodesis. Our current practice is to systematically perform biceps tenodesis to the pectoralis major, given the high incidence of LHB enlargement, inflammation, and spurring of the inter-tubercular groove encountered intra-operatively.

Subscapularis dysfunction following TSA manifests as pain, instability and a lack of active maximal internal rotation, and is associated with poorer outcomes [31, 32]. There have been numerous studies aimed at determining the best way to address the subscapularis tendon, with the main options being mid-substance tenotomy, subscapularis peel and lesser tuberosity osteotomy (LTO) [31, 33-35]. A mid-substance tenotomy is carried out one centimeter medial to the lesser tuberosity [36]. Miller **et al*.* [31] identified abnormal subscapularis function in 28/41 (68.2%) of patients who underwent TSA with mid-substance subscapularis tenotomy and repair, manifested as difficulty tucking their shirt into the back of the pants, and confirmed clinically using both the lift-off and belly-press tests. An ultrasound study performed by Jackson **et al*.* [37] identified 7/15 repairs as ruptured six months after mid-substance subscapularis tenotomy repair in TSA, and found significantly worse internal rotation strength, and Disabilities of the Arm, Shoulder, and Hand (DASH) scores in this group.

The LTO as originally described by Gerber **et al*.* consists of elevating a small fragment of the lesser tuberosity (5 to 10 mm in thickness, and 3 cm in length) with the subscapularis insertion using an osteotome [38]. This approach has generally yielded good results, with greater than 80% of patients achieving the ability to tuck in a shirt behind their back post-operatively [39, 40]. When comparing LTO with tenotomy in a retrospective series of 36 shoulders treated with shoulder arthroplasty for primary OA, Jandhyala **et al*.* [36] reported better strength and clinical outcomes in the LTO group. Subscapularis peel involves elevating the tendon off the lesser tuberosity from lateral to medial, beginning at the inter-tubercular groove [41]. Recent publications have compared LTO with subscapularis peel in TSA. A retrospective cohort analysis of 60 patients treated with TSA for shoulder OA with either LTO (28 patients) or subscapularis peel (32 patients) demonstrated statistically insignificant differences in the belly-press test, bear-hug test, Western Ontario Osteoarthritis of the Shoulder (WOOS), DASH, and Constant scores between the cohorts at minimum one year follow-up. Lapner **et al*.* [41] conducted a prospective multicenter double-blind randomized controlled trial comparing LTO with subscapularis peel in TSA, and found no difference in subscapularis strength, or clinical outcomes (as measured by a handheld dynamometer, the WOOS, and ASES scores) at two year follow-up. Their follow-up CT study also showed no difference in tendon healing and fatty infiltration rates between the two groups [42].

#### 3.1.2 Subscapularis-Preserving Approach

This approach begins with a vertical incision beginning just anterior to the posterior margin of the acromioclavicular joint, followed by a superficial dissection down to deltoid fascia, with full-thickness skin flaps developed on both sides of the incision to allow for access to the anterior, and posterior borders of the acromion [43]. The deltoid is then split between the anterior and middle raphe, and subperiosteally elevated off the acromion. Exposure of the rotator interval is then done by placing a Hohman retractor medially at the level of the coracoid process. Next, the interval is opened, and the humeral insertions of the coracohumeral and superior glenohumeral ligaments are detached, followed by the coracoid, and glenoid insertions, respectively. The rotator interval flap is then retracted posteriorly over the supraspinatus, and the resulting exposure extends from the anterior border of the supraspinatus tendon to the anterior border of the supraspinatus footprint.

Lafosse **et al*.* [43] treated 22 consecutive patients with primary TSA through the superior approach, 17 of which were included in the analysis at two years. Postoperative rehabilitation consisted of immediate unrestricted active, and passive range of motion with a physical therapist. All patients demonstrated significantly improved postoperative functional outcome scores and range of motion compared to preoperative, and there were no signs of radiographic complications. Their results compared favorably to other similar studies utilizing the deltopectoral approach. Subscapularis function was preserved in all, as confirmed by the belly-press test. Furthermore, the authors hypothesize better soft-tissue tensioning using this approach, since the glenohumeral joint is never dislocated, thus resulting in less soft-tissue disruption. However, they did note that 6 patients had humeral head malpositioning, 8 had residual inferior osteophytes, and 5 had an improperly-sized humeral head, which may be explained by the limited exposure through this approach.

Ding **et al*.* [44] recently published short-term radiographic results of an ongoing prospective randomized trial comparing primary TSA through the deltopectoral and subscapularis-sparing approach to a traditional approach. Surrogate measurements for anatomic humeral head restoration were humeral head height, head centering, medial offset, humeral head diameter (HHD), and head-neck angle. At six weeks, there were no significant differences in any of the above parameters between the two groups. However, the subscapularis-sparing group showed a significantly higher number and size of residual postoperative osteophytes. The authors highlight a lack of evidence linking residual osteophytes to clinical outcomes.

#### 3.1.3 Anteromedial Approach

The anteromedial approach to the shoulder dates back to the early 20^th^ century [45], but it was only later described in the context of shoulder arthroplasty by Neer **et al*.* [13, 46]. This approach involves making an incision 1 cm lateral to the coracoid tip, extending along the anterior aspect of the deltoid toward its insertion on the humerus, releasing the entire origin of the anterior deltoid from the clavicle to the anterior-most aspect of the acromion, retracting the deltoid laterally, and developing a plane proximal to distal [47]. The deltoid is then reattached to its origin with non-absorbable, transosseous sutures. Gill **et al*.* [47] reviewed 75 patients who underwent shoulder arthroplasty through the anteromedial approach, and found no significant anterior deltoid weakness post-operatively compared to pre-operative strength testing. Foruria **et al*.* [48] reviewed 723 consecutive shoulder arthroplasties, 110 of which were done through the anteromedial approach (15%). Interestingly, 39% of revision cases utilized this approach, compared to only 9.5% of primary cases. Furthermore, 77 of 110 anteromedially-approached shoulders (70%) had previous surgery, compared to only 110 of 613 (18%) of deltopectoral cases. While the deltoid healed to its insertion in all cases, this study was not able to rule out residual anterior deltoid weakness post-operatively. In difficult shoulder arthroplasty cases where increased exposure is desired, the anteromedial approach to the shoulder appears to be a reasonable option to use, keeping in mind the potential risk of residual anterior deltoid weakness.

## 4 HUMERAL PREPARATION

When preparing to resect the humeral head, adequate exposure is paramount, which may be achieved with the placement of a large Darrach retractor in the glenohumeral joint and blunt Hohmanns in the subacromial space and inferior neck [9]. This should be followed by removal of osteophytes to better expose the plane of the articular surface. This in turn determines the proper version and angle of the humeral osteotomy. Variable-angle systems, in which a freehand cut is made through the plane of the articular margin, are especially reliant on native neck anatomy exposure. If normal anatomical landmarks are absent, fixed-angle systems may be used which employ a cutting guide (intra- or extra-medullary) to determine the osteotomy site [49]. The osteotomy site can later be revised if needed following canal preparation, and the trial component itself may be used as a cutting guide. The version of the osteotomy is also determined by the plane of the articular margin, which ranges from 0° to 55° of retroversion [50]. However, determining the appropriate amount of retroversion intraoperatively can be very challenging when the anatomy is unreliable. Pre-operative templating with appropriate advanced imaging is highly recommended to avoid errors.

Restoring the anatomic humeral head size is important, as it has been shown that even small changes in head size can negatively affect shoulder biomechanics. For example, Harryman **et al*.* [51] demonstrated that increasing the humeral head thickness by 5 mm resulted in a 20° to 30° loss in range of motion. Decreasing the head size can also negatively impact outcome by reducing the surface arc for motion between the head and the glenoid, resulting in point loading on the glenoid, and early greater tuberosity (GT) impingement. Furthermore, anatomic positioning of the head is also critical; superior placement increases supraspinatus tension, inferior placement results in early GT impingement, anterior placement causes increased subscapularis tension, and early posterior impingement, while posterior placement causes increased tension on the rotator cuff, and early anterior impingement [49].

## 5 GLENOID BONE LOSS: TECHNICAL CHALLENGES

Proper glenoid preparation may be the most critical step in total shoulder arthroplasty since most causes of failure are attributable to glenoid-sided wear and loosening. Careful pre-operative planning is required in order to better understand the bony anatomy and adjust the surgical plan accordingly. The goals of glenoid preparation are to correct any abnormalities in version, while leaving behind enough bone to support the implant. The glenoid wear pattern classification by Walch **et al*.* [52] is the most widely used method of assessing the glenoid, and it provides crucial information that will guide the surgeon intraoperatively. In type A1, the humeral head is centered, and there is some glenoid erosion medially, while in type A2, the erosion is major. In type B1, the humeral head is subluxated posteriorly, but there is no erosion, while the type B2 glenoid (biconcave) demonstrates posterior erosion, as well as posterior subluxation. In type C, the glenoid is severely retroverted (>25°), but not necessarily worn posteriorly [53].

Types B2 and C glenoids are the most challenging to treat, and require a different approach than the usual concentric reaming method of glenoid preparation. The goal is to restore neutral glenoid version [54], as failure to do so increases the shear loads across the humerus and can lead to early implant loosening, and failure [55, 56]. However, when faced with severe posterior-sided wear or a dysplastic glenoid, overcorrection may have a negative effect. This can occur with compromise of the available bone stock for glenoid implant placement and decreasing the soft-tissue tensioning on the implant leading to instability [53]. In such cases, restoring the glenoid to within 10° of the patient’s native anatomy may be a better option, although accurately predicting this position is a challenge in its own right, even with advanced imaging [57].

**Fig. (3):**
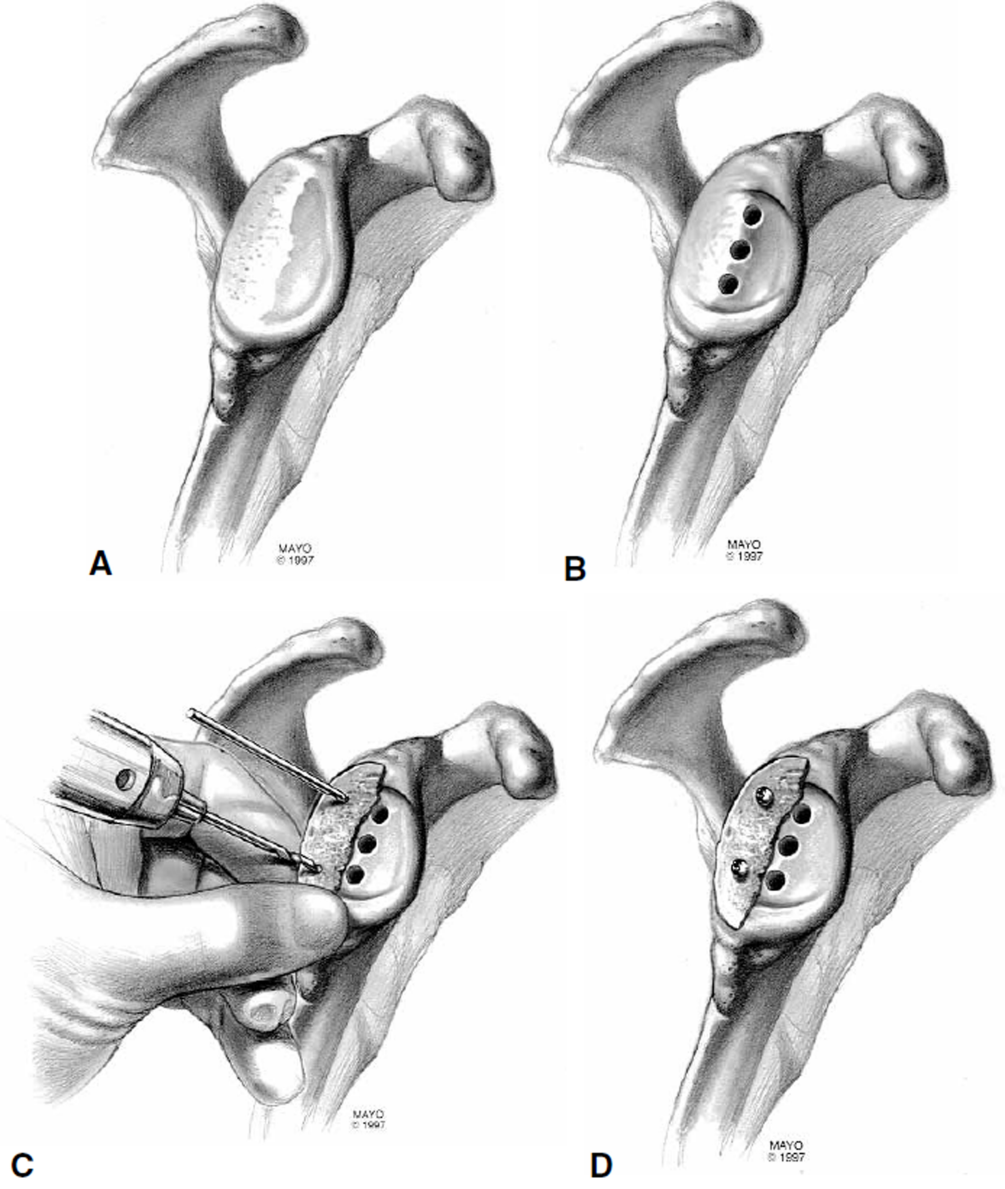
Illustration depicting steps in glenoid bone grafting. **(A)** Glenoid with significant posterior erosion. **(B)** Preparation of the anterior glenoid. **(C)** Bone graft placement in deficient area, temporarily held in place with drills. **(D)** Replacement of the drills with 3.5 mm cortical screws. (Adapted from Steinmann SP, Cofield RH. Bone grafting for glenoid deficiency in total shoulder replacement. Journal of shoulder and elbow surgery / American Shoulder and Elbow Surgeons [*et al*] 2000;9:361-7.).

There are three main options to choose from when faced with a type B2 or C glenoid: asymmetric reaming, posterior bone grafting, and posteriorly-augmented implants. In the case of posterior erosion less than 1 cm and retroversion less than 15°, the anterior glenoid can be reamed eccentrically to off-set this deformity. Corrections greater than 15° should be avoided since they may violate the glenoid vault, resulting in implant penetration upon insertion [58-60]. When the deficiency exceeds 1 cm, and the glenoid is retroverted more than 25°, bone grafting with internal fixation should be used, although this technique has had mixed results [53, 61-63]. Neer **et al*.* [61] described excision of the worn area of the glenoid down to bleeding bone and insertion of a pre-contoured corticocancellous humeral head autograft to fill the defect. The graft is then secured by means of cortical screws, inserted either anteriorly or posteriorly, or by wedging the graft into the glenoid medial to the defect like a keystone, and securing it with non-absorbable sutures (Fig. **3**). A sequential approach involves preparing the high side first, followed by placement of column holes, provisional fixation of the humeral head graft on the low side with K-wires or 2.5 mm drill bits, and ending with 3.5 mm cortical screw insertion, which are countersunk so as to not interfere with the implant [63]. Between 15° and 25° of retroversion, implants with posterior augments may result in better clinical outcomes and fewer complications when compared to bone graft although there remains little evidence to support this recommendation [64]. However, despite these technical advances, the rates of glenoid component loosening, residual posterior humeral component subluxation, and shoulder instability remain high for the type B2, and C glenoid treated with TSA [6, 65].

## 6 REVERSE TOTAL SHOULDER ARTHROPLASTY FOR THE TYPE B2 GLENOID

More recently, Mizuno **et al*.* [66] described the use of semiconstrained RTSA in patients over the age of 70 with type B2 glenoid deformities that were not amenable to correction with conventional asymmetric reaming of the high side of the glenoid. Conceptually, they felt that the screw fixation of the glenoid baseplate in might provide better stability in comparison to the cemented polyethylene glenoid component of the TSA. Their study reviewed twenty-seven patients with primary glenohumeral OA who underwent RTSA for a biconcave glenoid deformity, ten of whom required adjunctive bone grafting due to severe posterior wear, and all of whom had intact rotator cuffs. The mean pre-operative retroversion was 32°, and mean subluxation of the humeral head relative to the axis of the scapula was 87%. The surgical technique consisted of asymmetric reaming of the glenoid to achieve <10° of retroversion, and 10° of inferior tilt, followed by fixation of the glenoid baseplate with either four or five screws, depending on the amount of available bone stock. If the retroversion could not be corrected to <10°, or if >20% of the baseplate was unsupported after reaming, autogenous humeral head or iliac crest bone graft was used. The results were promising, with significant postoperative improvements in mean Constant scores, and shoulder range of motion, and a 93% overall patient satisfaction rate at a mean final follow-up of fifty-four months. The RTSA also prevented the recurrence of posterior humeral component subluxation in all cases. While more high quality studies are required to aid in establishing specific guidelines for the use of RTSA in patients with primary glenohumeral OA with severely deficient glenoids, it appears to be a promising option.

## 7 SUMMARY AND RECOMMENDATIONS

Shoulder arthroplasty can be a life-altering procedure for patients suffering from arthritis of the shoulder, and success rates are very high with few complications. In order to achieve successful results and maximize implant longevity, careful preoperative planning is paramount, and should be individualized to each patient’s clinical and radiographic presentation. Advanced imaging is essential, especially when faced with a deformed glenoid. Glenoid preparation may be complex in the case of significant retroversion and posterior erosion. Eccentrically reaming the high side of the glenoid is usually safe in the case of mild posterior erosion and retroversion, but overcorrection places the residual bone stock at risk of failure. When the deformity is severe, the available options include bone graft augments, or implant augments, although clinical results are mixed. A promising novel approach that may improve clinical outcomes involves using RTSA to treat severe glenoid deformities, although the surgical indications for the use of this technique require further study.
